# Assessing beauticians’ knowledge of cutaneous melanoma and willingness to contribute to melanoma surveillance practices on the general population^☆^^☆☆^

**DOI:** 10.1016/j.abd.2020.03.016

**Published:** 2020-09-15

**Authors:** Laura Vollono, Giovanni Paolino, Anna Buonocore, Michele Donati

**Affiliations:** a Dermatology Unit, Tor Vergata University, Rome, Italy; b Dermatology Clinic, La Sapienza University, Rome, Italy; c Dermatology Unit, Scientific Institute San Raffaele, Milan, Italy; d Department of Pharmacy and Cosmetology, University of Siena, Siena, Italy; e Surgical Pathology, Campus Biomedico University, Rome, Italy

Dear Editor,

Melanoma it is the most aggressive skin cancer, with a high mortality rate. Delayed diagnosis contributes to the poor prognosis. Early detection is the best approach to improve the outcome in melanoma patients.^1^, ^2^, ^3^

Approximately 50% of melanomas go undetected at self-inspection.^4^ To avoid the consequences of delayed diagnosis, all medical and non-medical professionals who have direct contact with the patients’ skin can play a pivotal role in correctly referring patients to a dermatologist in case of suspicious lesions. Beauticians observe a great extent of the skin of their clients, and therefore have remarkable access for detecting melanocytic lesions. Moreover, beauticians are used to be in contact with their clients, especially women, more often than dermatologists or family physicians. This study aimed to investigate melanoma knowledge, surveillance practices, and attitudes among beauticians in the metropolitan Rome area, and to raise awareness to the potential role of beauticians in early detection and referral of cutaneous melanoma.

Data were obtained through self-administered questionnaires completed by 30 certified beauticians working at salons in Rome, Italy, during a one-day symposium conducted by a dermatologist (L.V.), designed to illustrate the basic clinical parameters for melanoma detection and raise awareness about melanoma screening. The questionnaire was inspired by that used in the study by Roosta et al., who first investigated head and neck melanoma surveillance practices and attitude among hairdressers.^2^ It consisted of two different parts: part 1 was administered before the symposium, in order to investigate current knowledge and behavior of the participants; part 2 was administered after the symposium, in order to investigate the extent of knowledge acquired during the lecture, the expected future behavior, and the willingness to learn more about melanoma screening and to refer to dermatologists.

A 100% response rate was obtained. All beauticians were female, with a mean age of 37 years. The majority of them were unclear about the correct signs of melanoma. Nevertheless, an overwhelming majority of beauticians (96.7%) desired to learn more about melanoma detection (Fig. 1). The most preferred educational modalities were live symposiums (50%) and videos (40%). Many beauticians already examined their clients’ skin, regardless of being asked to do so. When a suspicious lesion was detected, most beauticians (90%) referred their client to seek attention from a physician. A significant proportion of referrals resulted in some form of medical diagnosis (90%). Of note, almost all beauticians (96.7%) reported that they would be willing to inform clients about an observed lesion after being properly trained in melanoma detection.

**Figure 1:**
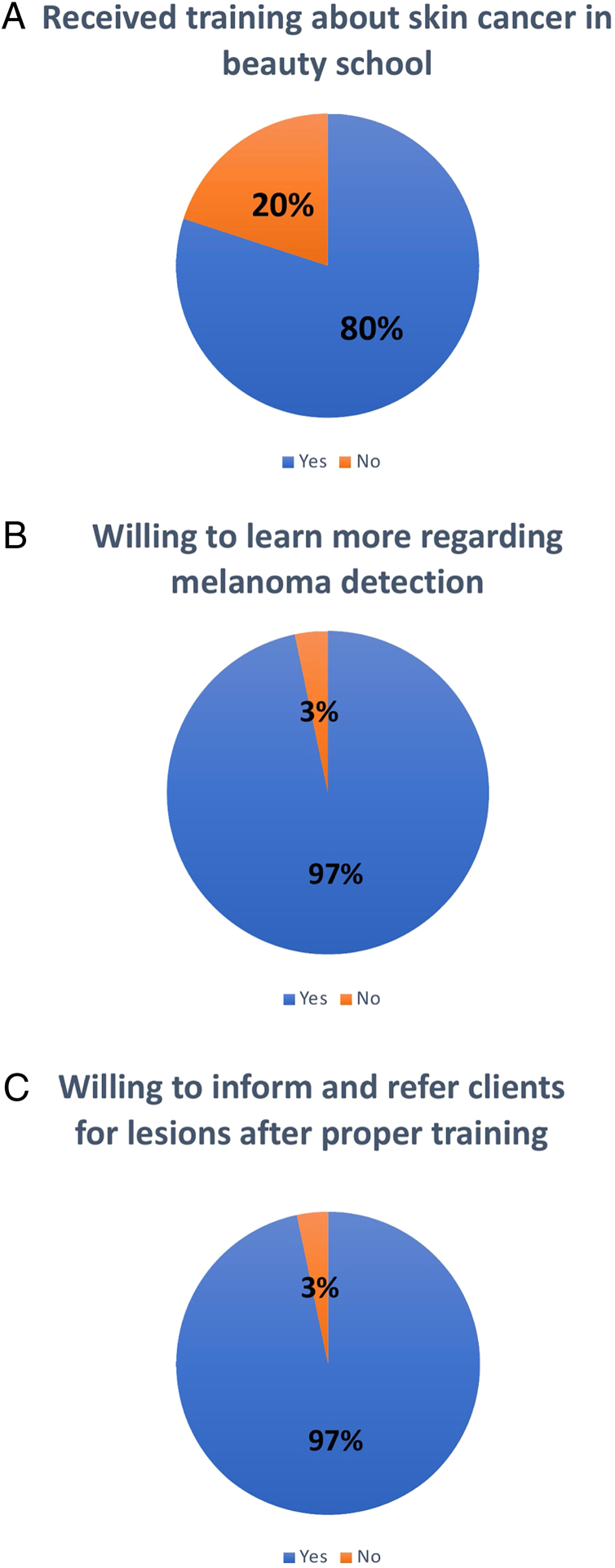
Percentage of beauticians who received training about skin cancer at beauty school (A), beauticians who are willing to learn more about melanoma detection (B), and percentage of beauticians who are willing to inform and refer clients to dermatologists for suspected lesions after receiving proper training (C).

To the best of the authors’ knowledge, this is the first study to investigate the potential role of beauticians in the detection of cutaneous melanomas in the general population. It follows a current research line that aims to identify new professional figures that may act as a bridge between dermatologists and the general population, improving melanoma’s secondary prevention.^5^ Indeed, similarly to hairdressers, beauticians are at a unique position to detect skin cancers, given their routine contact with a significant portion of the public and their close observation of body sites that patients are not normally able to observe, such as genitals or the back. However, this survey did not take into account the anatomical distribution of lesions. Another limitation of the present study is the small sample of beauticians included.

European law, in addition to defining beautician rules, establishes the parameters necessary for professional qualification, with specific training courses for the professionals. With this letter, the authors would like to raise awareness about the potential usefulness of properly trained beauticians in melanoma screening in the general community, and propose that dermatological modules should be mandatory in their training.

## Financial support

None declared.

## Authors' contributions

Laura Vollono: Approval of the final version of the manuscript; design and planning of the study; drafting and editing of the manuscript; collection, analysis, and interpretation of data; effective participation in research orientation; critical review of the literature; critical review of the manuscript.

Giovanni Paolino: Approval of the final version of the manuscript; effective participation in research orientation; intellectual participation in the propaedeutic and/or therapeutic conduct of the studied cases; critical review of the literature; critical review of the manuscript.

Anna Buonocore: Approval of the final version of the manuscript; design and planning of the study; drafting and editing of the manuscript; collection, analysis, and interpretation of data; effective participation in research orientation; intellectual participation in the propaedeutic and/or therapeutic conduct of the studied cases; critical review of the literature; critical review of the manuscript.

Michele Donati: Approval of the final version of the manuscript; design and planning of the study; drafting and editing of the manuscript; intellectual participation in the propaedeutic and/or therapeutic conduct of the studied cases; critical review of the literature; critical review of the manuscript.

## Conflicts of interest

None declared.
